# Solar Thermo-coupled Electrochemical Oxidation of Aniline in Wastewater for the Complete Mineralization Beyond an Anodic Passivation Film

**DOI:** 10.1038/s41598-018-21473-z

**Published:** 2018-02-15

**Authors:** Dandan Yuan, Lei Tian, Zhida Li, Hong Jiang, Chao Yan, Jing Dong, Hongjun Wu, Baohui Wang

**Affiliations:** Institute of New Energy Chemistry and Environmental Science, College of Chemistry and Chemical Engineering, Northeast Petroleum University, Daqing, 163318 P.R. China

## Abstract

Herein, we report the solar thermal electrochemical process (STEP) aniline oxidation in wastewater for totally solving the two key obstacles of the huge energy consumption and passivation film in the electrochemical treatment. The process, fully driven by solar energy without input of any other energies, sustainably serves as an efficient thermoelectrochemical oxidation of aniline by the control of the thermochemical and electrochemical coordination. The thermocoupled electrochemical oxidation of aniline achieved a fast rate and high efficiency for the full minimization of aniline to CO_2_ with the stability of the electrode and without formation of polyaniline (PAN) passivation film. A clear mechanism of aniline oxidation indicated a switching of the reactive pathway by the STEP process. Due to the coupling of solar thermochemistry and electrochemistry, the electrochemical current remained stable, significantly improving the oxidation efficiency and mineralization rate by apparently decreasing the electrolytic potential when applied with high temperature. The oxidation rate of aniline and chemical oxygen demand (COD) removal rate could be lifted up to 2.03 and 2.47 times magnification compared to conventional electrolysis, respectively. We demonstrate that solar-driven STEP processes are capable of completely mineralizing aniline with high utilization of solar energy. STEP aniline oxidation can be utilized as a green, sustainable water treatment.

## Introduction

Aniline is an important raw material for various chemical processes, widely used in the production of polyurethanes, rubber additives, dyes, pharmaceuticals, pesticides, herbicides and other industries^1–3^. Due to the high toxicity and tough biodegradability, wastewater containing aniline needs to be treated before discharging into the aquatic environment to protect public and environmental health^4,5^. Many advanced technologies have been researched and developed for the treatment of aniline wastewater by focusing on physical methods, such as adsorption methods^6,7^ and membrane separation technologies^8^, chemical oxidation such as advanced oxidation processes (AOPs) by photocatalytic degradation^9–11^, photoassisted Fenton oxidation^12^ and electrochemical treatment^13^, biological methods such as the natural degradation and biodegradation^14^, and combinations of the above methods^15^. Accordingly, the electrochemical treatment of wastewater is one of the greener and more promising treatments. However, it is a very costly process—a cost efficiency of 1000 mg-COD kWh^−1^ was estimated for the direct anodic oxidation of wastewater^16–18^.

Two methods were performed for the electrochemical treatment of aniline wastewater. The first used a high voltage for breaking up the passivation film, but required huge energy consumption and heavy side water splitting. The other was operated at low voltage while a thick passivation film was formed which resisted sustainable oxidation. So, these current studies have clearly demonstrated that the electrochemical treatment of aniline wastewater has two principal obstacles: the first is the huge energy consumption needed, and the other is the electrolytic production of polyaniline, that is, passivation film, which significantlyhinders further electrolysis and application. Therefore, research on the electrochemical treatment of aniline wastewater has been focused on the fast and sustainable oxidation and digestion of passivation film sat the appropriate electrolytic potential. Many studies have looked at the fabrication of the anode for the natural oxidation of the passivation film^19,20^. Nevertheless, few improvements have been advanced for stopping the passivation film.

In this paper, a complete solution to solve these two key obstacles at the same time, called the “STEP oxidation of aniline wastewater,” is presented. A key settlement to the first problem is to switch the convention to use renewable resources, such as the sustainable solar energy. A comprehensive solution to the second issue is to control the formation of the passivation film under the relative low potential, which uses the well-known electrochemical oxidation of aniline as a key control step to quickly digest the passivation film.

The rapid growth of public awareness about the importance of environmental health has induced researchers to introduce advanced technology that reduces the utilization of fossil fuels for the emission of pollutants. Solar energy has been considered to be the green fuel for the twenty-first century. In our previous research, the solar thermal electrochemical process (STEP)^21–23^ has been demonstrated to work successfully for efficient chemical reactions such as the STEP iron^24^, ammonia^25^, CO_2_ capture-to-fuel^26,27^, organic synthesis^28,29^, and recent STEP coal conversion by using solar thermo- and electrochemistry to minimize the amount of fossil energy required^30^, while maximizing the rate of electrolysis reactions by lowering the electrolysis potential.The STEP process and chemistry were detailed in our recent paper^31^. This process effectively utilizes solar energy to drive the chemical reaction process by: first, converting all the energy from the solar source towards the chemical reactions; and second, the cooperative and coupling use of the solar photo-, thermo-, and electrical fields for chemical reactions. In this way, the balance of the conversion (solar to three energy fluxes) and utilization (chemical reaction) effectively improves the reaction efficiency and selectivity. Herein, we design and investigate a novel and intrinsic method for a vast improvement of aniline oxidation beyond the passivation film for the full and sustainable mineralization on the basis of the characteristics of the targeted pollutant and STEP chemistry. In this paper, we focus on applying this new technology for the effective treatment of aniline wastewater and totally solving the two key obstacles. As a novel adaptation and adoption of the STEP process, the effective joining of solar thermochemistry can make the electrochemical potential significantly reduced for some reactions, especially endothermic oxidation reactions, thus actually reducing the electrochemical energy.

In the paper, the STEP aniline treatment successfully completed the degradation of aniline, wherein solar energy is the sole power source without any other forms of energy input. The goals were set as “solar utilization” and “moving beyond an anodic passivation film.” The results exhibited that the stop of the PAN passivation film or the rapid dissolution substantially lifted the reaction rate and mineralization rate under the combined action of thermochemistry and electrochemistry. It was considered a sustainable process for the treatment of the aniline wastewater in both the energy utilization and continuous aniline oxidation without the formation of passivation film. A novel and energy-free way for wastewater treatmentwas accomplished by the synergistic use of solar energy without any other input of energy.

## Experimental Section

### Chemicals and Materials

All aqueous chemical solutions were prepared with deionized water. Sources of the main reagents include aniline (C_6_H_7_N, 99.5%, Shenyang Xinxi Chemical,), absolute ethyl alcohol (C_2_H_6_O, 99.5%, Henyang Huadong Chemical,), sulfuric acid (H_2_SO_4_, AR, Shenyang Chemical) and sodium sulfate (Na_2_SO_4_, AR, Tak Sun Chemical).

### Indoor Experimental Device

The configuration of the integrated STEP reactor was fabricated by a glass of the sealed and heatable device (100 mL) equipped with a heating unit and electrolysis unit. The electrolysis unit was composed of the aniline-containing electrolyte and electrodes. Both the anode and cathode were made of platinum foil with a size of 2 × 2 cm^2^.

The simulated wastewater containing aniline was prepared by adding 200 mg/L aniline to the aqueous solution according to the composition of actual industrial sewage. The supporting electrolyte of Na_2_SO_4_ was added to the aniline wastewater for ready electrolysis.

### Outdoor Experimental Device

**Figure 1:**
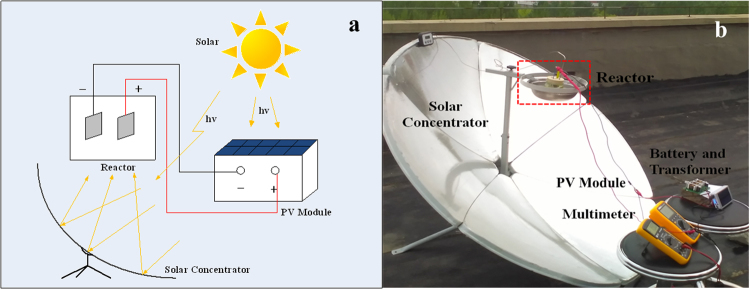
The outdoor experimental setup of the STEP aniline oxidation.

The outdoor experimental apparatus was composed of three parts: a photoelectric, a photothermal, and a electrochemical unit (Thermo-electrochemical Reactor) for the investigation of the STEP aniline oxidation. Theschematic diagram is shown in Fig. 1(a and b), and the details were describedin our previous papers^32,33^.

A paraboloidal solar concentrator (1.5 m, Max. Temp. 500 °C) was used for the solar photothermal units at highmagnification equipped with a 2-dimensional tracking reflector system to control the temperature of the reactor by moving the receiver to intersect this line. A polycrystalline silicon solar module (solar cell, 3 W/12 V,VUAVA, China) was used for the solar photoelectric unitsfor the oxidation and storage with a rechargeable Li-ion battery and voltage regulator. The STEP thermo-electrolytic energy is extracted from those two units.

The thermo-electrochemical reactor consisted of a two-electrode electrochemical cell supplied by the solar electrounit and thermounit. The reactor with the inlet from a wastewater reservoir and outlet to dischargewas centered on the focus point of a paraboloidal solar concentrator. The wastewater was pumped through the reservoir to the reactor. A regulator was used for voltage and current control of the cell voltage. The reaction time of the oxidation in the reactor was adjusted by a flow rate for the stationary state/flow state (returned to the reservoir for recycling) experiments.

The real aniline wastewater was sampled from Petrochina (Jilin). The data were recorded as 239.5 mg/L of aniline and 1487.9 mg/L of chemical oxygen demand (COD).

### The Analytical Method of the STEP Aniline Oxidation

According to the accepted definition of chemical oxygen demand (COD), COD analysis was employed herein for detection of the mineralized degree of aniline with both carbon and nitrogen elements. The aniline and intermediates were monitored by a UV-vis spectrophotometer during the oxidation of aniline in the aqueous solution.

For each sample, the aniline and intermediates were determined with UV-vis spectrophotometry (Shimadzu UV-1700) in an interval sampling. The COD was measured by the standard potassium dichromate trimetric method. The test of the thermo-dependent cyclic voltammograms (CV) was scanned by using BAS Epsilon-EC electrochemical workstation with three electrode systems including the working electrode and counter electrode of the platinum electrode (10 mm × 10 mm), and the reference electrode of thesaturated calomel electrode (SCE) at a sweep rate of 10 mV/s.

## Results and Discussion

Many papers have extensively reported the chemistry of most relevant methods for the oxidation of organic pollutants. The mechanisms involve some chemical processes: breakdown (degradation/decolorization) of the pollutant molecules (A), production of the intermediates (B or C, D), and mineralization of both pollutants and intermediates to carbon dioxide (CO_2_) in a chemical mode,

1 $${\rm{A}}\to {\rm{B}}\,\mathrm{and}/\mathrm{or}\,{\rm{C}}\to {\rm{D}}\to {{\rm{CO}}}_{{\rm{2}}}.$$

The A to CO_2_ undergoes either an indirect production of intermediates (B, C, or D) or a direct mineralization. A direct conversion of pollutant molecule to CO_2_ favors the process of the mineralization.

In this case, the oxidation of aniline is generally matched by the following co-reaction,

2 $${\rm{AN}}\to {\rm{PAN}}\,\mathrm{and}/\mathrm{or}\,{\rm{B}}\to {{\rm{CO}}}_{2}+{{{\rm{NO}}}_{3}}^{-}.$$

The breakdown/degradation of organic pollutants during electrochemical treatment is usually monitored by the disappearance of primary pollutants given by Equation 3:

3 $${\rm{Degradation}}\,( \% )=[{{(\mathrm{ABS}}_{0}}^{{\rm{M}}}-{{{\rm{ABS}}}_{{\rm{t}}}}^{{\rm{M}}}{{)/\mathrm{ABS}}_{0}}^{{\rm{M}}}]\times {\rm{100}}{\rm{.}}$$

where ABS_0_^M^ and ABS_t_^M^ are the absorbences of a targeted molecule (M) before and after an oxidation time *t*, respectively, at the maximum absorption wavelength of the pollutant molecule determined by UV-vis spectrophotometry. The substitution of pollutant concentration by ABS^M^ in the equation is a common misunderstanding ofan indirect process of the intermediates (B, C, D), whereas it is suitable for the direct mineralization.

The mineralization process of organic pollutants can directly be monitored from the decay of their COD related to the oxidation of organics consisting of C, N, and S elements.

From these fundamentals, our two systems have been built up to clearly probe this procedure for the investigation of the degradation of the starting pollutant, production of the intermediates, and mineralization of the organics via the theoretical and experimental demonstrations.

### Theoretical Calculation of the STEP Aniline Oxidation

The STEP oxidation of aniline can occur directly through electron transfer between the anode and cathode. Eqs(4–6) summarize the electrode reaction of full mineralization of aniline by the STEP chemical oxidation of aniline to CO_2_ and NO_3_^−^ in the anode (4) and H_2_ evolution in cathode (6),

For the anode, electrochemical oxidation of aniline to carbon dioxide and nitrate radical:

4 $${{\rm{C}}}_{6}{{\rm{H}}}_{5}{{\rm{NH}}}_{2}({\rm{l}})+15{{\rm{H}}}_{2}{\rm{O}}({\rm{l}})\to 6{{\rm{CO}}}_{2}({\rm{g}})+{{\rm{HNO}}}_{3}+36{{\rm{H}}}^{+}({\rm{l}})+36{e}^{-}.$$

For the cathode, hydrogen evolution:

5 $$36{{\rm{H}}}^{+}({\rm{l}})+36{e}^{-}\to 18{{\rm{H}}}_{2}({\rm{g}})$$

Full cell reaction:

6 $${{\rm{C}}}_{{\rm{6}}}{{\rm{H}}}_{{\rm{5}}}{{\rm{NH}}}_{{\rm{2}}}({\rm{l}})+15\,{{\rm{H}}}_{2}{\rm{O}}({\rm{l}})\to 6{{\rm{CO}}}_{2}({\rm{g}})+{{\rm{HNO}}}_{3}+18{{\rm{H}}}_{2}({\rm{g}}).$$

By the calculation of the electrooxidation potential, the input and coupling of the solar heat can reduce the energy required for the electrolysis process. These processes can be determined using the data for available entropy S, enthalpy H, and free-energy G, and they are identified by a negative isothermal temperature coefficient of the cell potential^34^. The isothermal coefficient (dE/dT)_isoth_ can be derived from the electric potential of a constant temperature cell^23,35^:

7 $${(\mathrm{dE}/\mathrm{dT})}_{{\rm{isoth}}}={\rm{\Delta }}{\rm{S}}/{\rm{nF}}=({\rm{\Delta }}{\rm{H}}-{\rm{\Delta }}{\rm{G}})/{\rm{nFT}}$$

At any electrolysis temperature of T_STEP_, and at the unit activity, the electrolytic reaction has an electrochemical potential of E°_T_. The value can be obtained by using fixed thermodynamic data, as:

8 $${\rm{E}}{^\circ }_{{\rm{T}}}=-{\rm{\Delta }}{\rm{G}}^\circ ({\rm{T}}={{\rm{T}}}_{{\rm{STEP}}})/\mathrm{nF};\,\,\,{\rm{E}}{^\circ }_{{\rm{ambient}}}={\rm{E}}{^\circ }_{{\rm{T}}}({{\rm{T}}}_{{\rm{ambient}}})$$

Here,

9 $${{\rm{T}}}_{{\rm{ambient}}}=298{\rm{K}}=25\,^\circ {\rm{C}}{\rm{.}}$$

In Eq. (8), E°_T_ is the standard electrode potential of the electrochemical reaction. The standard electrode potential can judge whether an electrochemical reaction occurs spontaneously. If E°_T_ > 0, the reaction can occur spontaneously without external electric field; while if E°_T_ < 0, there action is non-spontaneous.

From the calculation of E°_T_, ΔG^◦^ can also be achieved through the Gibbs-Helmholtz equation, as follows:

10 $${\rm{\Delta }}{\rm{G}}^\circ ={\rm{\Delta }}{\rm{H}}^\circ -T{\rm{\Delta }}{\rm{S}}^\circ $$

For a non-standard ΔH condition, it needs to be calculated:

11 $${\rm{\Delta }}{\rm{H}}^\circ ={{\rm{\Delta }}{\rm{H}}^\circ }_{298}+{\int }_{298}^{{\rm{T}}}{{\rm{\Delta }}{\rm{C}}}_{{\rm{p}}}{\rm{\Delta }}\mathrm{dT}$$

12 $${\rm{\Delta }}{\rm{S}}^\circ ={\rm{\Delta }}{\rm{S}}{^\circ }_{298}+{\int }_{298}^{{\rm{T}}}{{\rm{\Delta }}{\rm{C}}}_{{\rm{p}}}/\mathrm{TdT}$$

Here:

13 $${{\rm{\Delta }}{\rm{C}}}_{{\rm{p}}}={\sum }_{i}{{\rm{n}}}_{{\rm{i}}}{{\rm{\Delta }}{\rm{C}}}_{{\rm{pi}}}.$$

**Figure 2:**
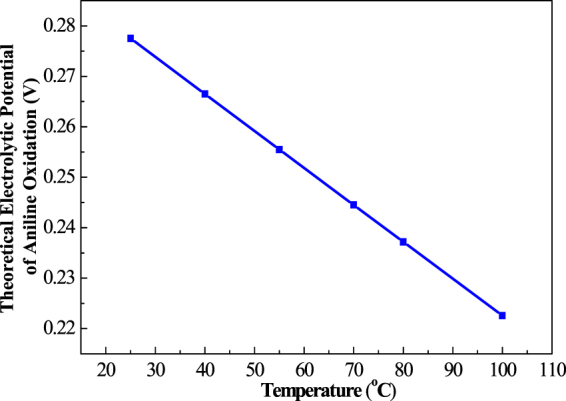
The temperature variation of theoretical electrolytic potential of STEP aniline oxidation.

From the 298 K data of the reactants and products, we get the integral constant value of ΔH°_298_, so for any standard enthalpy change ΔH°, for any Gibbs energy ΔG°, we finally obtain standard electrode potential E°_T_ under arbitrary temperature. Because E_T_ = −E°_T_, from the electrolytic cell reaction we can get the potential. For the case of complete electrochemical degradation of aniline in the cell reaction, thermodynamic data can be obtained from Table 1 by referring to the reaction in the reference materials under different temperatures. These data can be calculated to get the cell reaction ΔH° and ΔG°, so as to obtain the theory of electrode potential. The variation of the aniline electrolysis potential with temperature is calculated with Eq. (8) and presented in Fig. 2. The curve demonstrates that the aniline oxidation potential decreases with increasing temperature. For example, from 25 °C to 100 °C, the aniline electrolysis potential in the aqueous phase decreases from 0.2794 V to 0.2221 V. The data means that the STEP process can be applied favorably to aniline electro oxidation aided by a little heat. This decline provides a theoretical basis for the STEP process for effective and efficient mineralization of aniline beyond a passivation film on the anode.

**Table 1:** Thermodynamic data and calculations of the thermo- and electrochemical oxidation of aniline.

T (K)	C_p.m_ (J·mol^−1^·K^−1^)					△C_p_ (kJ·mol^−1^·K^−1^)	△_r_H°_m_ (kJ/mol)	△_r_S°_m_ (kJ·mol^−1^·K^−1^)	△_r_G°_m_ (kJ/mol)	E°_T_ (V)	E_T_ (V)
	C_6_H_7_N	H_2_O	CO_2_	H_2_	HNO_3_						
298	168.8	75.32	40.20	28.84	110.1	−0.4282	1721	2.541	963.7	−0.2775	0.2775
313	172.9	75.20	40.61	28.93	109.8	−0.4268	1715	2.521	925.6	−0.2665	0.2665
328	174.8	75.24	41.03	29.00	109.6	−0.4278	1708	2.502	887.5	−0.2555	0.2555
343	181.1	75.38	41.45	29.06	109.5	−0.4307	1702	2.484	849.4	−0.2445	0.2445
353	183.9	75.53	41.73	29.09	109.4	−0.4334	1697	2.473	824.0	−0.2372	0.2372
373	189.4	75.92	42.29	29.14	109.5	−0.4404	1688	2.452	773.1	−0.2226	0.2226

The basic thermodynamic data cited from NIST Chemistry WebBook.

### Indoor Study of the STEP Aniline Oxidation

Based on the theory of the STEP process, the utilization of more solar heat and less electricity results in a higher solar conversion efficiency due to the present technological status of the high solar-to-thermal conversion and low solar-electro conversion. It is well known that decreasing the electronic energy of an endothermic reaction by increasing the temperature provides energy savings or that keeping the potential at high temperature will increase the electronic energy for a powerful reaction. In the STEP aniline oxidation, a high temperature is provided just by excess and non-costly solar heat.

In our study, STEP aniline oxidation was affected by many factors and conditions such as the reaction temperatures, times, initial concentrations, pH value, etc. We focused on four domains for investigations of the reaction conditions.

**Figure 3:**
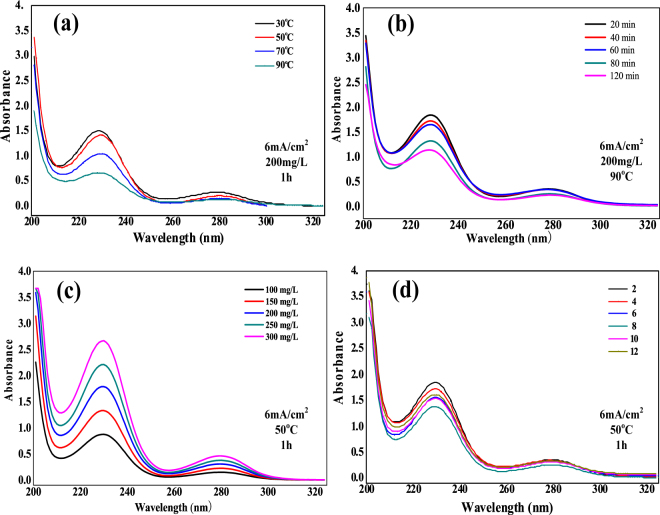
UV spectra and absorbances of the STEP aniline oxidation at different (**a**) reaction temperatures, (**b**) reaction times, (**c**) initial concentrations, and (**d**) pH.

As shown in the Fig. 3, the maximum absorption wavelength of the aniline molecule is determined to be 230 nm by UV-vis spectrophotometry. The degradation of the starting aniline can be calculated by using Eq. (3).

**Figure 4:**
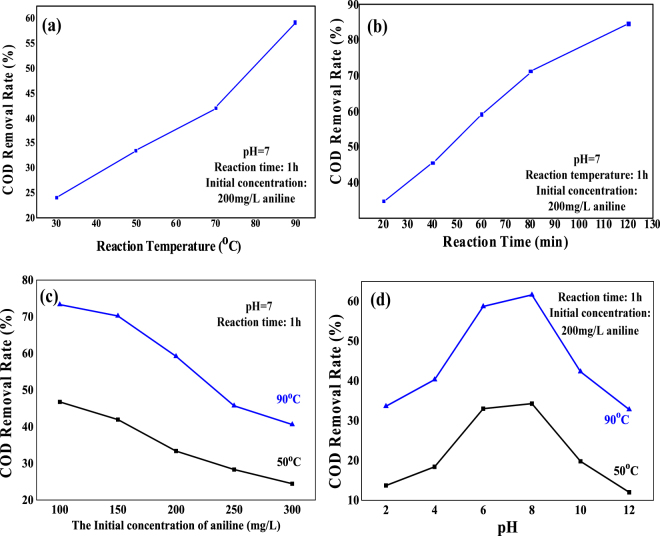
The COD removal rate of the STEP aniline oxidation at different (**a**) reaction temperatures, (**b**) reaction times, (**c**) initial concentrations, and (**d**) pH.

Following the STEP aniline oxidation, the corresponding mineralization was measured by the COD monitoring as displayed in Fig. 4.

Four variables were tested to confirm their role in the solar thermo- and electrochemical combination featured by the STEP aniline oxidation. The decay of COD/absorbance in Figs 3 and 4(a,b) illustrates that the STEP aniline degradation/on factors of temperature (heat) and time (stability), but not linearly with factors like initial concentration or pH.

The first observation was that COD/absorbance of aniline tends to decrease sharply with the increase of reaction temperature between 70 and 90 °C, which is attributed to a jointing and coupling of solar thermochemistry (Fig. 4(a)). The second result showed a steady increase in the aniline mineralization with increasing reaction time. When the reaction time was 120 min, the COD rate of aniline being stably removed was about 84.6% Fig. 4(b). The test of the initial concentration of aniline proved that the oxidation efficiency was stabilized at a high temperature even at high concentrations, where polymer passivation films are easily formed (Fig. 4(c)). Finally, the pH curve gives a good example for the optimal conditions (pH = 6–8) which would not require an adjustment of pH range for the treatment (Fig. 4(d)).

**Figure 5:**
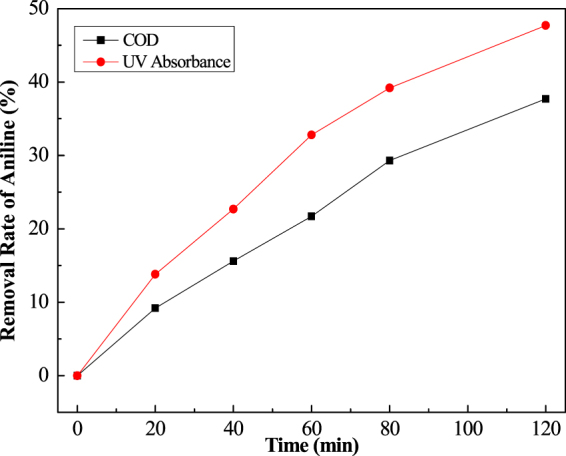
A comparison of the degradation based on absorbance measurement or the mineralization based on COD measurement (at 30 °C).

**Figure 6:**
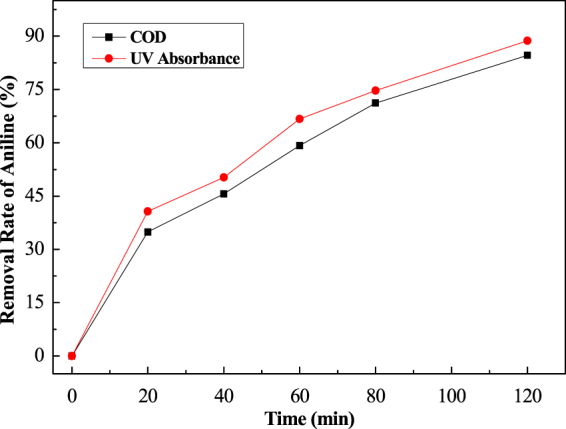
A comparison of the degradation based on absorbance measurement or the mineralization based on COD measurement (at 90 °C).

Two curves were plotted in Figs 5 and 6 for a comparison of the degradation expressed by the absorbance with the mineralization indicated by the COD.

The COD monitored the mineralization to CO2 and NO_3_^−^. The absorbance measurement values parallel the disappearance of aniline to the intermediates (COD existing) or CO_2_ and NO_3_^−^. Compared with Figs 5 and 6, the difference of the removal rate in both the COD and absorbance measurements indicated the appearance of intermediates, such as the passivation film (polymer) and small molecules. In the high temperature range, the small difference showed that no or few intermediates were created in the process.

The difference in the removal rates underscores the existence of the intermediates produced in the oxidation process at various temperatures, COD removal rate gets59.2% at 90 °C. It is a small difference, but a big value under the 30 °C conditions. It can be seen from Fig. 6 that the increase of the reactiveheatis in favor ofthe oxidation of aniline. It stops a production or performs a fast dissolution of the intermediates via a direct oxidation to CO_2_ + NO_3_^−^.

### Outdoor Study of the STEP Aniline Oxidation

For testing and verifying the thermo-electrochemical coupling effect and stability, fixed-volume flow experiments were performed at constant temperature and current in the outdoor facility with the simulated and real aniline wastewater. A 200 ppm solution of the simulated aniline wastewater was pumped into the sealed and heatable reactor with an in-time sampling at 30 °C and 90 °C. The COD removal rate of aniline is plotted versus reaction time.

**Figure 7:**
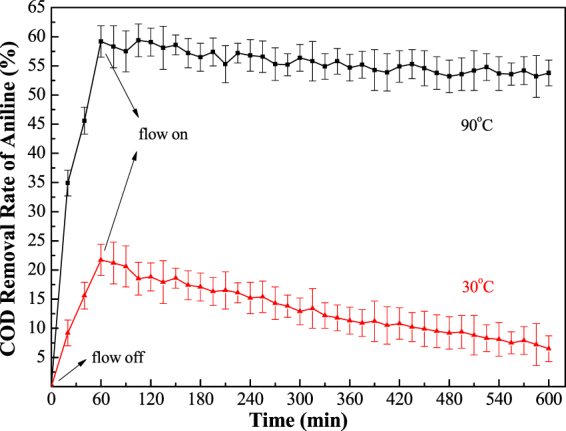
Outdoor tests of the STEP oxidation of the simulated aniline wastewater.

**Figure 8:**
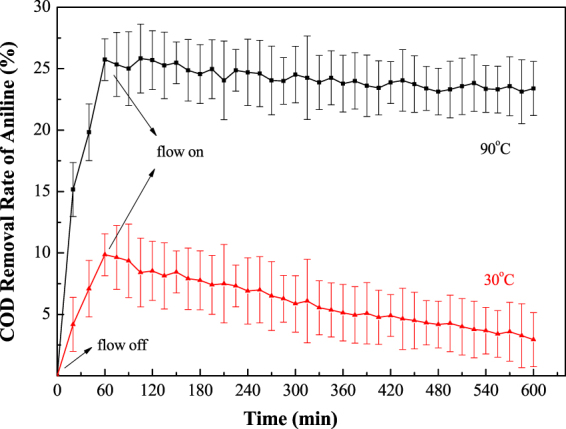
Outdoor tests of the STEP oxidation of the real aniline wastewater.

As shown in Figs 7 and 8, the removal rates of COD in both simulated and real aniline wastewater were kept constant with small variations for 10 hours at 90 °C while a low removal rate and highvariation occurred at 30 °C. Thus, the STEP process can achieve a fast and stable thermo coupled electrochemical oxidation. After 10 hours of electrolysis, the anode surface was checked, and was observed to be smooth and clean.

### Analysis of the Kinetics of the Aniline Oxidation

The thermo-electrochemical oxidation of aniline can occur directly in water to produce carbon dioxide and hydrogen, also generating nitrate radical in the absence of complex intermediates. The principle anode, cathode, and overall reactions occurring during the oxidization of aniline and reduction of water are summarized by Eqs (4)–(6):

For the overall reaction at 30 °C and 90 °C:

14 $$-\frac{{{\rm{dc}}}_{\mathrm{tAN}}}{{\rm{dt}}}=-\frac{{\mathrm{dA}[\mathrm{COD}]}_{{\rm{t}}}}{{\rm{dt}}}={\rm{kt}}{\rm{.}}$$

With variables defined as follows:

c_tAN_ = concentration of aniline at the time *t*;

[COD]_t_ = COD value at the time *t*;

[COD]_t_ = COD value at the time 0;

A = conversion coefficient of COD (as detailed at the beginning of section 3)

So,

15 $$-\frac{{\rm{d}}{[{\rm{COD}}]}_{{\rm{t}}}}{{\rm{t}}}={\rm{k}}^{\prime} {\rm{t}}$$

16 $${\rm{In}}\frac{{[\mathrm{COD}]}_{0}}{{[\mathrm{COD}]}_{{\rm{t}}}}={\rm{kt}}$$

**Figure 9:**
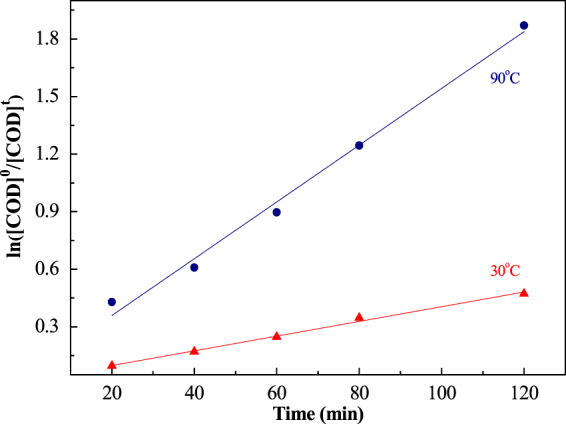
The first-order kinetics of aniline oxidation.

Figure 9 shows the first-order kinetics of aniline oxidation at different temperatures. The apparent constant *k* and correlation coefficient are presented in Table 2.

**Table 2:** Kinetics of aniline oxidation at different temperatures.

Temperature, °C	Apparent rate constant, *k*	Correlation coefficient, R^2^
30	0.0038	0.9928
90	0.0148	0.9887

It is obvious that increased temperature leads to a huge enhancement in kinetic rate. The kinetic rate constant of 90 °C is 3.89 times the value at 30 °C, and is a fast and efficient process. Nowdays, a rapid STEP process is achieved by raising the temperature.

### Study of Pathway and Mechanism for the STEP Aniline Oxidation

**Figure 10:**
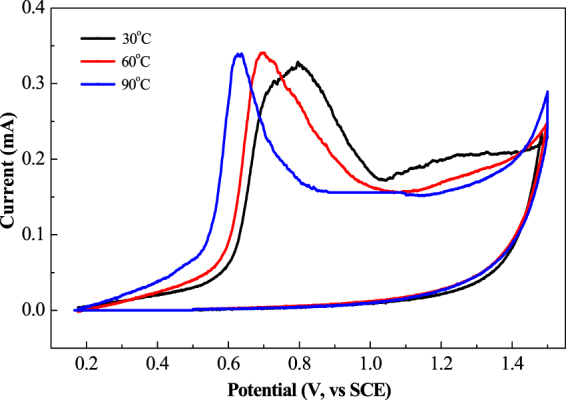
Thermo-dependent cyclic voltammograms of aniline solutions.

When trying to enhance solar energy utilization, it is necessary that the thermochemistry is perfectly combined with the electrochemistry. For an in-depth understanding of thermocoupled electrochemistry, the pathway and mechanism of the STEP oxidation reaction should be studied. The solar thermocoupled electrochemical effect can be displayed by using a measurement of thermo-dependent cyclic voltammetry (CV). The thermo-dependent CV curves were scanned as exhibited in Fig. 10.

The Fig. 10 shows the thermo-dependent CV of aniline at various temperatures. Two oxidation peaks appear in the oxidative direction, at 0.8 V and 1.2 V (vs. SCE), corresponding to the start of oxidation for aniline and intermediates polyaniline or/and benzoquinone. The oxidation potential of anilinewas decreased with the increase of temperature, while the oxidation peak of the polyaniline/benzoquinone gradually disappeared. This indicates that the temperature/heat can decrease the electrochemical oxidation potential of aniline, and lower the production of the intermediates, including the polyaniline. The oxidation of aniline was via intermediates to carbon dioxide at low temperatures, while the aniline was directly oxidized at higher temperatures. The temperature dependent CV results demonstrate that the thermocoupled electrochemistry switches reaction path ways from low temperatures to an alternative at high temperature with the effect of lowering potential and suppressing intermediates. Therefore, the thermo- and electrochemical oxidation of aniline can be accelerated by the STEP process beyond a passivation film on the anode for stable and full mineralization with added savings in energy consumption.

**Figure 11:**
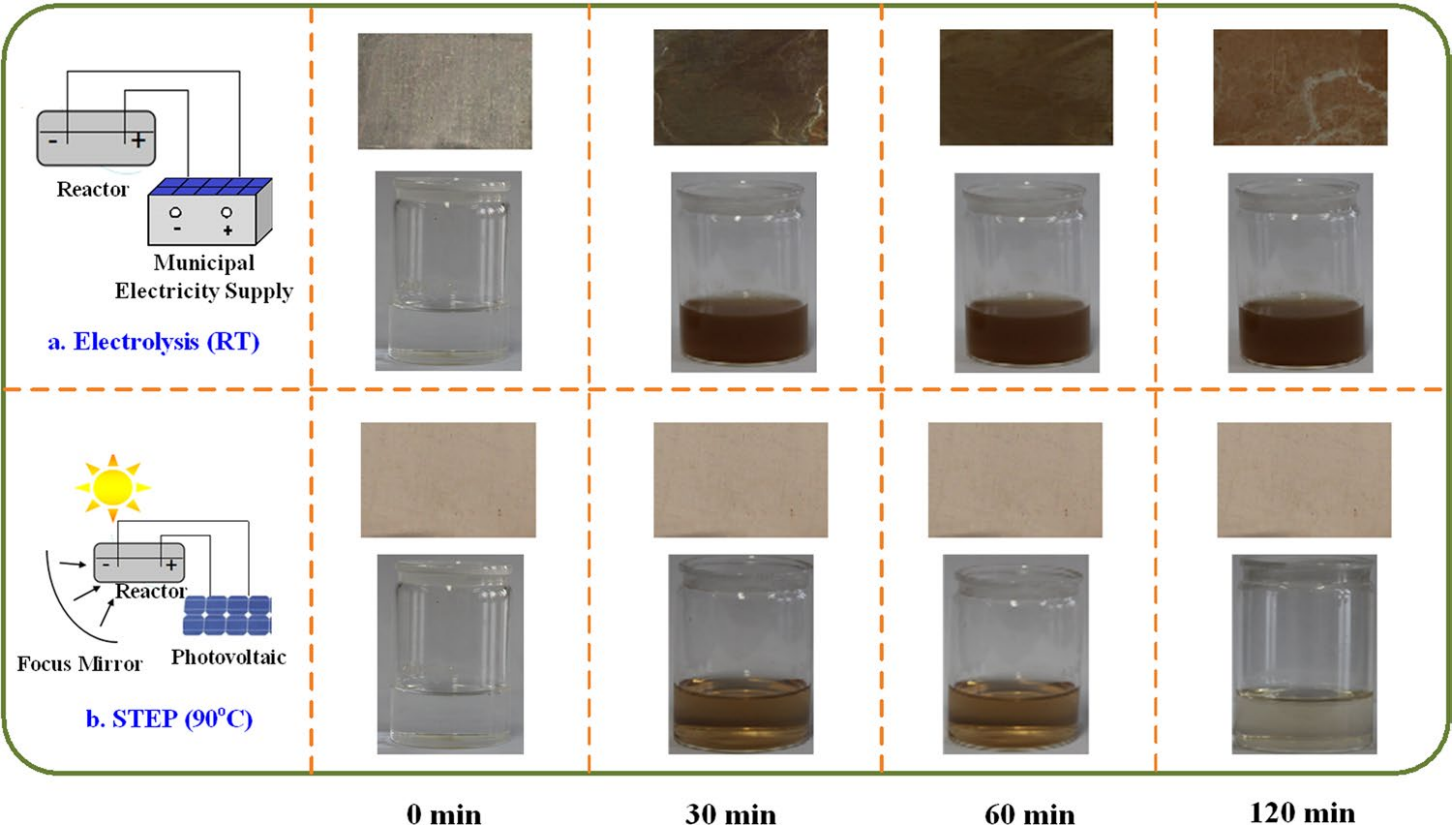
The images of variations of the anodic surface and aniline solution during the different oxidation processes.

Figure 11 shows the variations of the anodic surface and aniline solution during the different oxidation processes. From Fig. 11(a), it can be seen that the aniline solution gets dark brown with the formation of polyaniline (PAN) suspension in the room-temperature electrolysis, and the brown film, a polyaniline passivation film, was found to be formed on the anodic surface. With the increase of reaction time, the solution color and anode surface remained nearly unchanged. The dark suspension and brown film were identified as the polyaniline polymer and passivation film. Notably, the STEP oxidation at 90 °C (Fig. 11(b)) prevented the generation of PAN passivation materials on the anode and in the solution. This illustrates that a clean anode and clear solution were kept during the oxidation of 90 °C.

**Figure 12:**
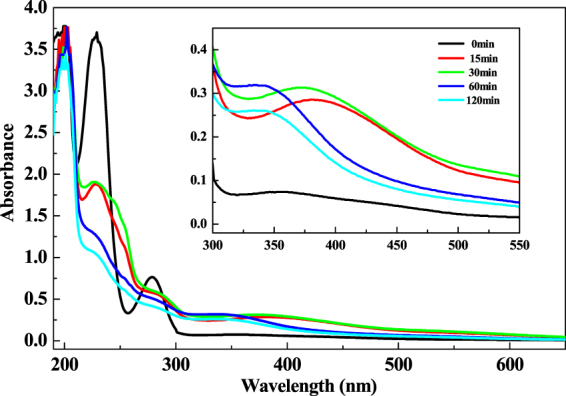
The UV-vis spectrum of aniline wastewater after room temperature electrochemical reaction with times.

Figure 12 presents the UV-visible spectra of the aniline electrochemical oxidation at different reaction times at room temperature. Many absorption peaks were found in the range of 200–500 nm, which were attributed to the intermediates, including the PAN, while only one peak from the starting aniline appeared in Fig. 3(b).With the existence of the passivation film, the oxidation of aniline stayed at a relatively low rate due to the many intermediates.

**Figure 13:**
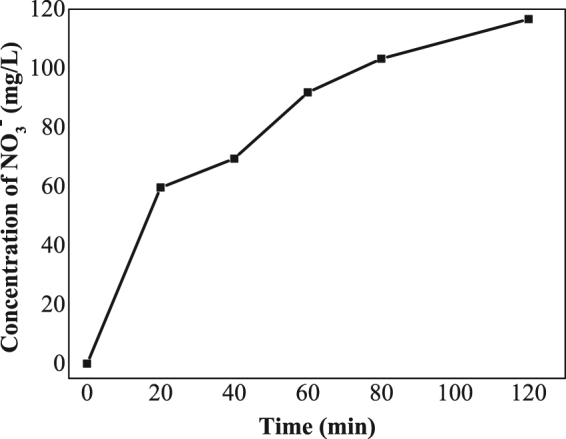
The variation of the NO_3_^−^ concentration during the STEP aniline oxidation at 90 °C.

The concentrations of NO_3_^−^ were also measured to prove the degradation of aniline, and the result is shown in Fig. 13. NO_3_^−^ concentration can achieve 116.6 mg/L after 2 hours of STEP aniline degradation at 90 °C. It is clear that a majority of aniline was oxidized through the STEP oxidation.

**Figure 14:**
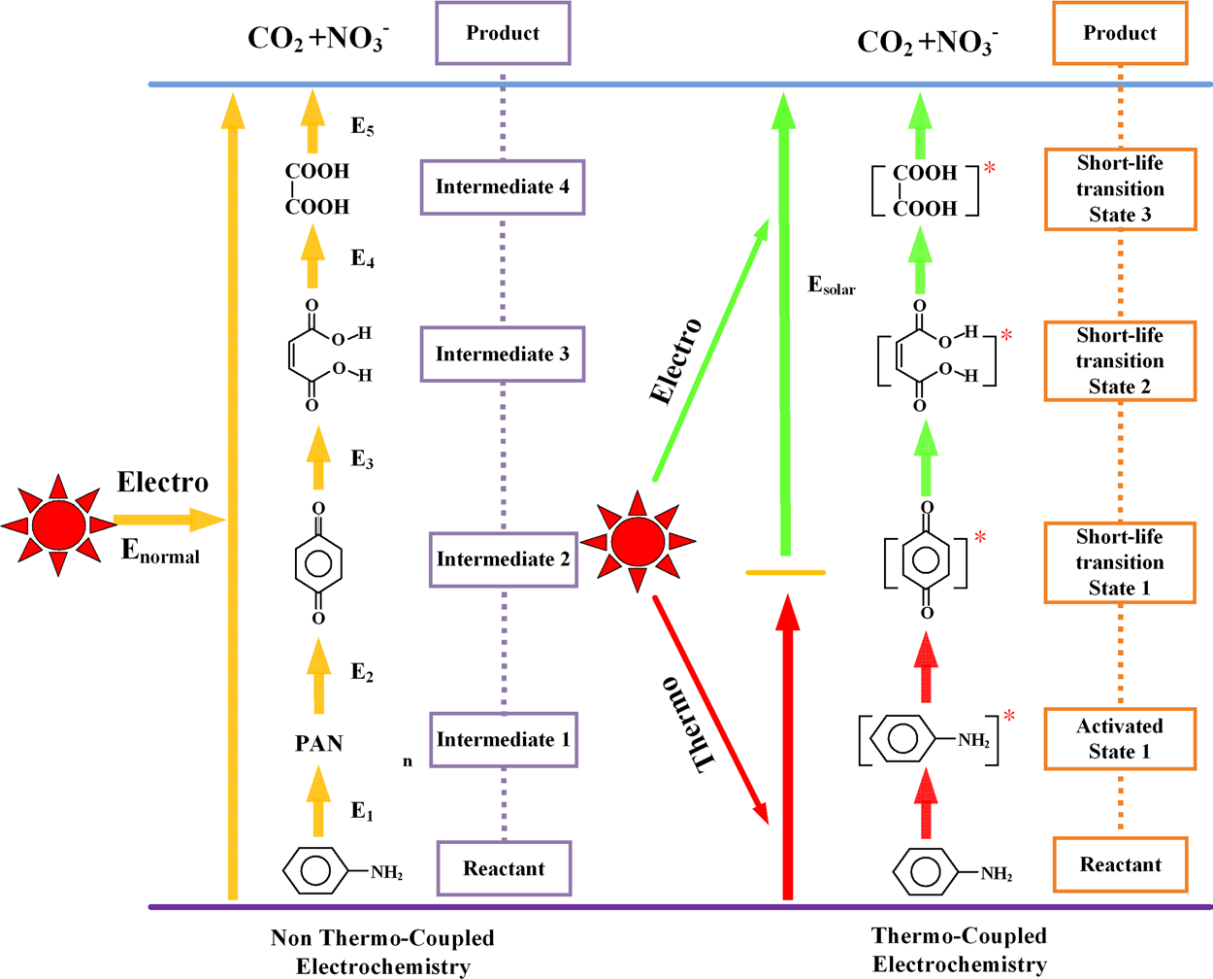
The proposed pathway and mechanism for the solar thermocoupled electrochemical oxidation of aniline.

Based on our experimental results, a proposed pathway and mechanism for the solar thermocoupled electrochemical oxidation of aniline are pictured in Fig. 14.

The electrochemical oxidation of aniline without the coupling of the solar heat undergoes the following four processes as shown in the left hand portion of Fig. 14, followed by the right side:

$${{\rm{C}}}_{{\rm{6}}}{{\rm{H}}}_{{\rm{7}}}{\rm{N}}\to {{\rm{C}}}_{{\rm{6}}}{{\rm{H}}}_{{\rm{4}}}{{\rm{O}}}_{{\rm{2}}}\to {{\rm{C}}}_{{\rm{4}}}{{\rm{H}}}_{{\rm{4}}}{{\rm{O}}}_{{\rm{4}}}\to {{\rm{C}}}_{{\rm{2}}}{{\rm{H}}}_{{\rm{2}}}{{\rm{O}}}_{{\rm{4}}}\to {{\rm{CO}}}_{2}+{{{\rm{NO}}}_{3}}^{-}+{{\rm{H}}}_{{\rm{2}}}{\rm{O}}{\rm{.}}$$

With the corresponding potentials being E_1_, E_2_, E_3_, E_4_, and E_5_, respectively. The right side of Fig. 12 provides a process to describe the STEP aniline oxidation. The activation energy of the aniline oxidation is decreased with increasing temperature. Aniline is then activated to the [Aniline]* state, and its intermediates exist (in some cases) in very short-life transition states at high reaction temperature. This reaction bypasses the intermediates to direct mineralization.

## Conclusions

This paper presents the novel solar STEP thermo-electrochemical process for the degradation of aniline wastewater beyond an anodic passivation film for stable and full mineralization. Via establishing the theory of a solar coupled thermal-electrochemical reaction of aniline wastewater, the reaction process and mechanism were analyzed theoretically and experimentally. The STEP oxidation of aniline achieved a fast rate and high efficiency for the full minimization of aniline in to CO_2_ while maintaining stability of the electrode and without formation of PAN passivation films. The experimental results show that the thermo-and electrochemical coordination passed over the appearance of the PAN passivation film during the reaction process on the surface of the anode. A clear mechanism of aniline oxidation was probed to determine a switch of the reactive pathway by theoretical analysis and experimental research. Due to the coupling of solar thermochemistry and electrochemistry, the electrochemical current kept stable, significantly improving the oxidation efficiency and mineralization rate by an apparent decrease in the electrolysis potential when applied with high heat. The system realized a faster reaction rate and higher efficiency, in which the oxidation rate and COD removal rate of aniline were lifted up to 2.03 and 2.47 times magnification than with conventional electrolysis, respectively. We demonstrated that the solar-driven STEP process was capable of completely mineralizing aniline with high utilization of solar energy. The STEP aniline oxidation fulfilled the goal of a green, sustainable water treatment. The broad application prospects of this method can significantly improve the efficiency of solar energy utilization and pollutant oxidation.
